# Supplementary material for: Combined Polarization/Magnetic Modulation of a Transverse NMR Gyroscope
Source: Sensors (Basel). 2023 May 11;23(10):4649. doi: 10.3390/s23104649 (PMC10222852; doi:10.3390/s23104649)
Supplement: Supplementary file 1 [file sensors-23-04649-s001.zip › sensors-2203728-supplementary.pdf]

Supplementary Materials: Combined Polarization/Magnetic Modulation of a Transverse NMR Gyroscope

Susan S. Sorensen [1], and Thad G. Walker [1]*

1. Matrix Inversion Least-Squares Fitting

If we expand Eq. 12 in Section 3.2, each term in the resulting expression will include one known factor and one unknown factor. Known variables include α^a, α^b, and θ_{PM}; unknown variables include A^S, A^a, A^b, C^S, C^y, C^{PD}, and the sine and cosine of $\delta^a - \epsilon_z$ and $\delta^b + \epsilon_z$. On the other side of the expression, S_z is our measured Faraday rotation signal. We can recast the expanded expression as a matrix equation, $S_z = M.b$, where M is a matrix containing all of the known functions, and b is a matrix containing the unknowns. We can solve this expression for b through matrix-inversion $b = (M^t M)^{-1} M^t S_z$.

Given that there are ten fit parameters, we can measure multiple points before taking a least-squares fit [1] to improve the goodness of fit. Through matrix inversion, we can solve for our unknowns at a series of discrete points and, then, make a least-squares fit to minimize the root mean square difference between the data and our fitting function. We chose to measure 50 points before performing our least-squares fit. The full matrix expression is then given in Eq. 1, where $\Theta = \text{sign}(\cos(\theta_{PM}))$, and subscripts $1, 2, ...50$ represent points taken at different times.

$$\begin{bmatrix} S_{z,1} \\ S_{z,2} \\ \vdots \\ S_{z,50} \end{bmatrix} = \begin{bmatrix} \sin(\alpha_1^a) & \cos(\alpha_1^a) & \sin(\alpha_1^b) & \cos(\alpha_1^b) & 1 & \sin(\alpha_1^a)\Theta_1 & \cos(\alpha_1^a)\Theta_1 & \sin(\alpha_1^b)\Theta_1 & \cos(\alpha_1^b)\Theta_1 & \Theta_1 \\ \sin(\alpha_2^a) & \cos(\alpha_2^a) & \sin(\alpha_2^b) & \cos(\alpha_2^b) & 1 & \sin(\alpha_2^a)\Theta_2 & \cos(\alpha_2^a)\Theta_2 & \sin(\alpha_2^b)\Theta_2 & \cos(\alpha_2^b)\Theta_2 & \Theta_2 \\ \vdots & & & & & & & & & \\ \sin(\alpha_{50}^a) & \cos(\alpha_{50}^a) & \sin(\alpha_{50}^b) & \cos(\alpha_{50}^b) & 1 & \sin(\alpha_{50}^a)\Theta_{50} & \cos(\alpha_{50}^a)\Theta_{50} & \sin(\alpha_{50}^b)\Theta_{50} & \cos(\alpha_{50}^b)\Theta_{50} & \Theta_{50} \end{bmatrix} \cdot \begin{bmatrix} C^S A^a \cos(\delta^a - \epsilon_z) \\ C^S A^a \sin(\delta^a - \epsilon_z) \\ C^S A^b \cos(\delta^b + \epsilon_z) \\ C^S A^b \sin(\delta^b + \epsilon_z) \\ C^S C^y + C^{PD} \\ A^S A^a \cos(\delta^a - \epsilon_z) \\ A^S A^a \sin(\delta^a - \epsilon_z) \\ A^S A^b \cos(\delta^b + \epsilon_z) \\ A^S A^b \sin(\delta^b + \epsilon_z) \\ A^S C^y \end{bmatrix} \quad (1)$$

References

1. Press, W.H.; Teukolsky, S.A.; Vetterling, W.T.; Flannery, B.P. *Numerical recipes in C (2nd ed.): the art of scientific computing*; Cambridge University Press: New York, NY, USA, 1992.

Disclaimer/Publisher's Note: The statements, opinions and data contained in all publications are solely those of the individual author(s) and contributor(s) and not of MDPI and/or the editor(s). MDPI and/or the editor(s) disclaim responsibility for any injury to people or property resulting from any ideas, methods, instructions or products referred to in the content.
